# Assessment of Fresh Homologous Osteochondral Transplantation in Knees as a Salvage Treatment: A Prospective Case Series with a Minimum Follow-up Period of 10 Months

**DOI:** 10.1055/s-0044-1792116

**Published:** 2024-12-21

**Authors:** Guilherme Gracitelli, Pedro Henrique Schmidt Alves Ferreira Galvão, Rayana Ueda Carrer, Fernando Cury Rezende, Marcelo Seiji Kubota, Carlos Eduardo Franciozi

**Affiliations:** 1 Departamento de Ortopedia e Traumatologia, Escola Paulista de Medicina, Universidade Federal de São Paulo, São Paulo, SP, Brasil; 2 Grupo de Joelho, Clínica Ortopédica Ortocity/Grupo H+ Brasil, São Paulo, SP, Brasil; 3 Departamento de Ortopedia, Traumatologia e Medicina do Esporte, Instituto Wilson Mello, Campinas, SP, Brasil

**Keywords:** allografts, cartilage, articular, knee/surgery, osteochondritis, transplantation homologous

## Abstract

**Objective** The present study evaluated the clinical outcomes and satisfaction of patients undergoing fresh homologous osteochondral transplantation in the knee as a salvage method.

**Methods** We analyzed eight knees from seven male patients who underwent fresh homologous osteochondral transplantation by a single surgeon. Their follow-up period ranged from 10 months to 5 years and 5 months. Clinical outcomes included the scores on the International Knee Documentation Committee (IKDC) and on the quality-of-life item of the Knee and Osteoarthritis Outcome Score (KOOS-QoL).

**Result** The sample consisted of complex cases since all operated knees had undergone previous surgeries. Functional improvement was variable, with six out of the seven operated patients showing statistically significant clinical improvement according to the IKDC score, and a single patient reported being moderately satisfied with the procedure. The quality-of-life item from the KOOS score improved in all patients. There was no failure, need for reintervention, or infection.

**Conclusion** Fresh homologous osteochondral transplantation is a safe salvage method in our setting to treat large lesions and those with failed previous procedures. Despite the small sample of this case series, most clinical outcomes were positive and had no complications.

## Introduction

Hyaline cartilage is an avascular tissue supplied by the diffusion of synovial fluid. 1 It has limited healing potential and low regenerative capacity. 2 Thus, the natural history of full-thickness articular cartilage lesions has poor outcomes, especially in young subjects. 3

Several reparative procedures stimulating bone marrow have been used to treat articular cartilage lesions, producing fibrocartilage tissue of inferior quality to the articular cartilage, 4 and their outcomes were lower in large lesions (> 3 cm ^2^ ). 5 For this reason, the ideal treatment for a focal cartilage lesion involves restoring the structure of the hyaline cartilage, 2 integrating the tissue into its periphery and the subchondral bone 4 to generate the lowest possible morbidity and a longer symptom-free period. 2

It is an “immune-privileged” tissue because its chondrocytes are embedded in an acellular matrix, with relative protection from the immune system. 6 7 In addition, it is non-neural structure, which makes it an ideal tissue for transplantation. 1

Fresh osteochondral transplantation allows treating extensive cartilage lesions and, in a single surgical procedure, restores the articular surface congruence with no donor site morbidity. 2 The objective of the current study was to evaluate the clinical outcome and satisfaction of a series of patients who underwent fresh homologous osteochondral transplantation in the knee as a salvage method with a minimum postoperative follow-up period of 10 months.

## Materials and Methods

After obtaining approval from the Ethics Committee, we identified seven patients, including one treated bilaterally (eight knees), who underwent fresh homologous osteochondral transplantation in the knee. This prospective study had a minimum postoperative follow-up period of 10 months. We assessed all patients before and after surgery using the International Knee Documentation Committee (IKDC) score and the quality-of-life item from the Knee and Osteoarthritis Outcome Score (KOOS-QoL). We applied the questionnaires to each patient preoperatively and then, at 6, 12, 24 months, and 5 years of postoperative follow-up.

The pairing between the recipient's and donor's knees used anteroposterior and lateral radiographs. 1 Donor tissues came from the tissue banks of Instituto Nacional de Traumatologia e Ortopedia (INTO) and F. E. Godoy Moreira from Hospital das Clínicas, Faculdade de Medicina, Universidade de São Paulo (HC-FMUSP). Because of the cellular viability of chondrocytes in the donor tissue, the transplant occurred up to 4 weeks after its collection and preparation, 4 These tissues were under refrigeration at 4 ^o^ C with no freezing 8 and no requirement for immunosuppressive therapy. 9

We indicated fresh osteochondral transplantation for osteochondral lesions > 2 cm ^2^ in diameter, classified as grade III or IV by the International Cartilage Repair Society (ICRS). The most common lesion was osteochondritis dissecans (OCD) in the medial femoral condyle (MFC). However, we also diagnosed MFC osteonecrosis (ON), patellar degenerative lesion resulting from patellofemoral instability (PFI), failure of a previous synthetic implant (SaluCartilage - SaluMedica, LLC, Atlanta, GA, USA) in the MFC and trochlea, and posttraumatic chondral lesion due to lateral tibial plateau fracture.

All patients operated on in this case series had presented failure of previous non-operative and surgical treatments in the knee, and we proposed fresh osteochondral transplantation as a salvage method to postpone the potential need for knee arthroplasty.

The procedure started after confirming the identification, conditioning, and quality of the allograft, which was kept in a saline solution until use. A medial parapatellar arthrotomy was the surgical technique performed in most cases. A single case of lateral plateau transplantation used the extended lateral parapatellar approach. After joint exposure, we debrided the edges of the lesion until reaching the subchondral bone. We measured the defect using a tool with cylindrical molds (the dowel technique) to determine the allograft size for harvesting.

The first case, with a follow-up period of 5 years and 5 months, was that of a 38-year-old male patient with a previous OCD diagnosis. The patient complained of pain in his left knee for 23 years and had undergone mosaicplasty and partial meniscectomy, with no significant improvement.

**Fig. 1:**
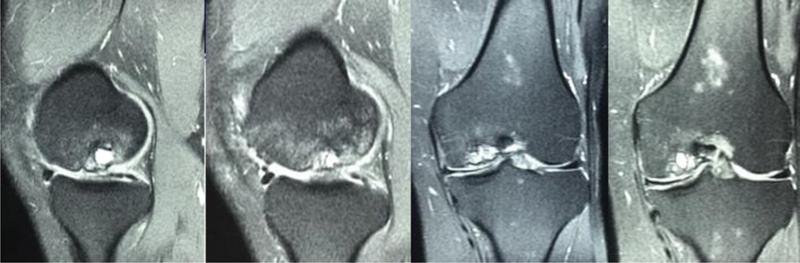
Coronal and sagittal magnetic resonance imaging sequences showing failure mosaicplasty integration of the medial femoral condyle and previous partial meniscectomy.

**Figs. 2:**
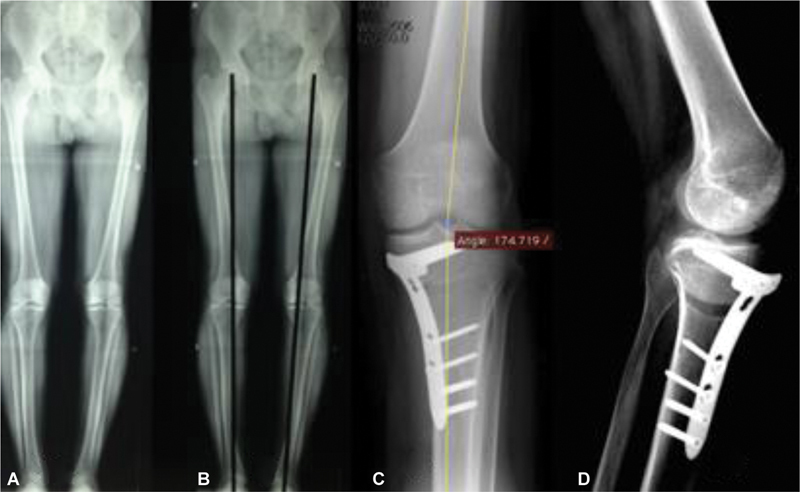
Panoramic radiographs of the lower limbs showing a slight mechanical axis in varus ( **A,B** ). Anteroposterior and lateral radiographs after valgus osteotomy with medial opening wedge and corrected final axis ( **C,D** ).

Fig. 1 shows the nuclear magnetic resonance imaging (MRI) from this patient, revealing the failure of the mosaicplasty with subchondral cysts in the MFC of the left knee. Figs. 2A–D illustrate the panoramic radiograph before osteotomy, with a traced mechanical axis showing slight varus, and the radiographs after valgus osteotomy with a medial opening wedge and corrected final axis.

**Figs. 3:**
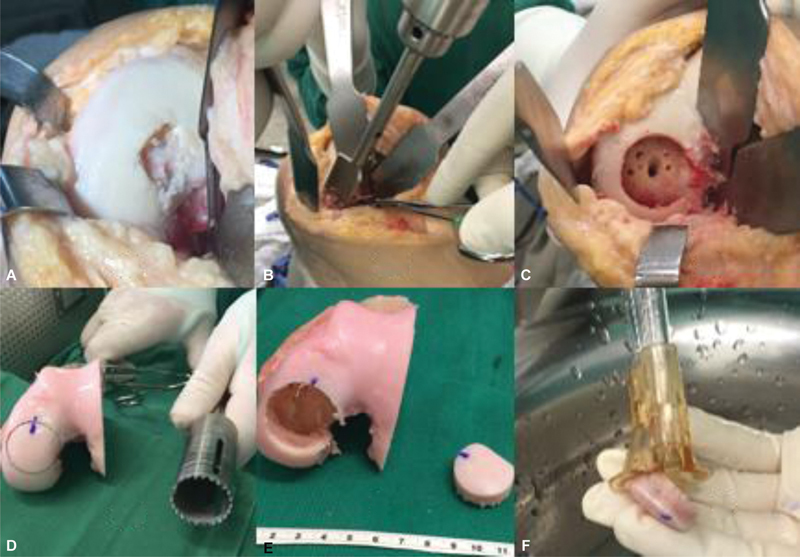
Intraoperative images showing the osteochondral defect before debridement ( **A** ), defect regularization with appropriate instrumentation ( **B** ), and the appearance after edge regularization ( **C** ). Image of the allograft ( **D** ), removal of the osteochondral plug with appropriate diameter according to previous measurement ( **E** ), and subsequent preparation and pulsatile lavage to remove the medullary components of the subchondral bone ( **F** ).

After arthrotomy and lesion identification ( Fig. 3A ), we debrided the defect, regularized it, and performed the proper measurements ( Figs. 3B,C ). Next, we prepared the allograft with its osteochondral plug ( Figs. 3D–F ).

We established the location for allograft removal by attempting to reproduce the site of the patient's lesion, using a guidewire through the center of the same cylindrical mold to center the hole saw for donor tissue removal. Maintaining perpendicularity to the articular surface, the depth of the plug beyond the cartilage (in the subchondral bone) was 3 to 4 mm, 1 and this same subchondral bone underwent a pulsatile lavage with saline solution to clean potential antigens from the donor tissue.

**Figs. 4:**
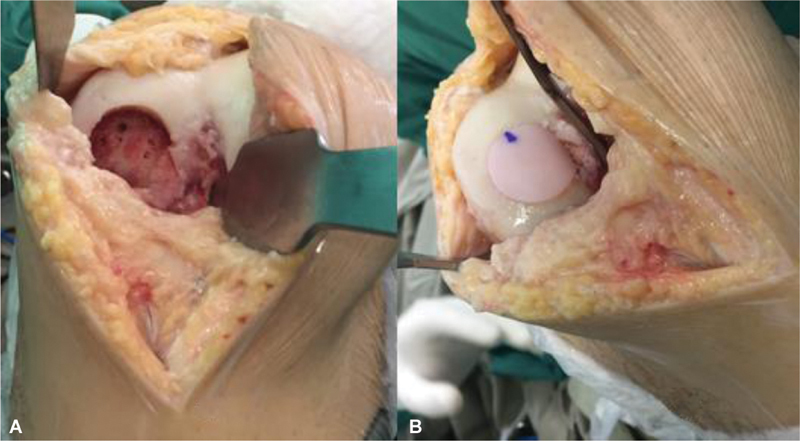
Intraoperative images before ( **A** ) and after plug fixation under pressure ( **B** ).

**Fig. 5:**
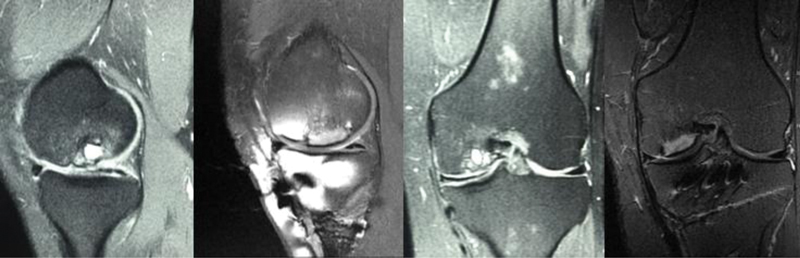
Coronal and sagittal magnetic resonance imaging sequences showing allograft integration one year postoperatively. The metal artifact consists of the fixation material of the osteotomy.

Figs. 4A,B shows the final presentation after intraoperative plug fixation and Fig. 5 depicts the follow-up MRI one year after surgery.

If the osteochondral lesion was larger than 25 mm in diameter, we removed more than one allograft plug with subsequent area overlapping (snowman technique). 1 Donor tissue fixation occurred under pressure (press-fit) and, if unstable, we added a Hebert screw in the center of each “unstable” plug to increase stability.

**Fig. 6:**
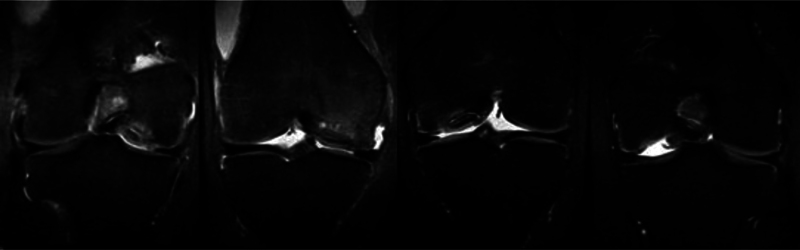
Coronal magnetic resonance imaging sequence showing the lesion in the medial femoral condyle of the right knee and left knee, respectively, resulting from osteochondritis dissecans.

The second case required a similar procedure, using two plugs in the left knee due to the extent of the lesion. This was a 21-year-old male patient with bilateral OCD and an unstable and loose osteochondral fragment in the joint ( Fig. 6 ). He presented bilateral genu varum and underwent valgus osteotomy in two stages.

**Figs. 7:**
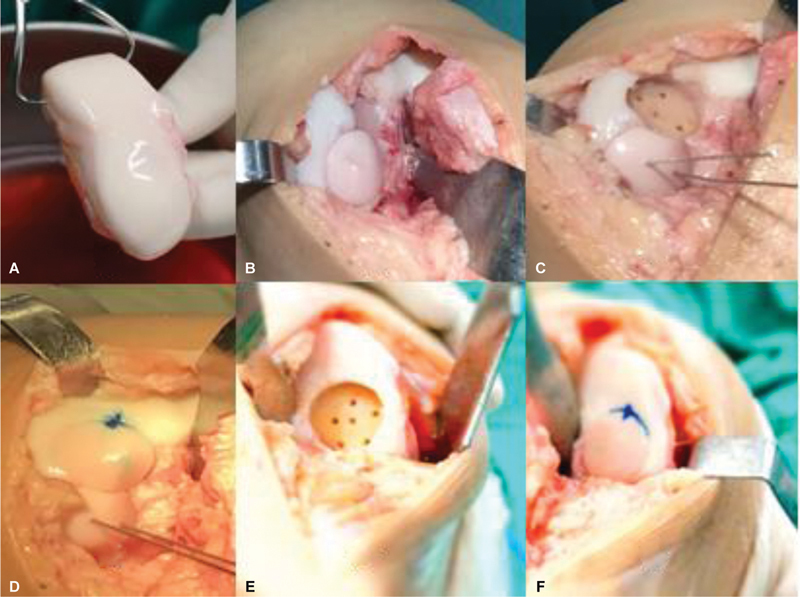
Fresh osteochondral tissue of the medial femoral condyle ( **A** ). Intraoperative photograph of the left knee during the fresh transplantation with two osteochondral plugs measuring 22.5 mm each ( **B,C** ), lower plug fixation with a headless screw, and upper plug fixation under pressure ( **D** ). Intraoperative photograph of the right knee during the fresh transplantation of the medial femoral condyle with a 22.5 mm osteochondral plug fixation under pressure ( **E–F** f).

**Figs. 8a–b:**
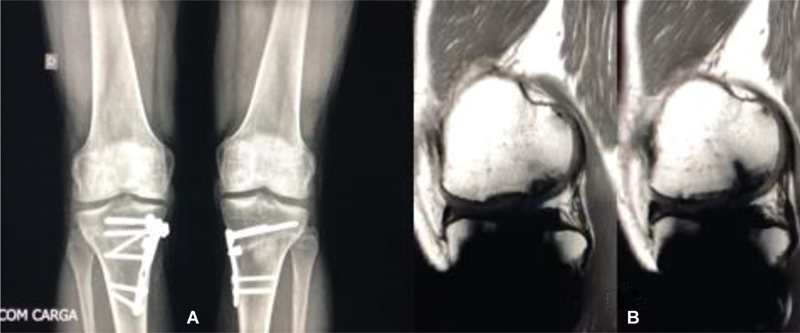
: Postoperative radiograph after the bilateral osteochondral transplantation with consolidated osteotomies (operated 5 months apart) ( **A** ). Postoperative T1-weighted magnetic resonance imaging of the right knee with metal artifacts 10 months after surgery. The images demonstrate good integration and adequate chondral surface ( **B** ).

Fig. 7 shows the intraoperative images of the osteochondral transplant. Figs. 7A–D depict the fresh donor osteochondral tissue and its preparation and fixation in the left knee with two plugs, one fixed under pressure and the second with a metal headless screw, as well as the surgical preparation of the right knee, performed 5 months after surgery on the left knee ( Figs. 7E,F ). The postoperative radiographic image ( Fig. 8A ) and MRI ( Fig. 8B ) 0 months after surgery demonstrate metal artifacts but good allograft integration and a uniform chondral surface.

The postoperative period included a rehabilitation protocol to protect the graft during incorporation into the recipient area. 10 We instructed the patients not to bear weight bearing on the operated limb for 6 weeks by using a long, articulated knee immobilizer in extension until quadriceps activation control. We restricted the range of motion to 90° in the first 4 weeks and encouraged quadriceps isometry during the immediate postoperative period and closed kinetic chain exercises after the sixth postoperative week. Sports activities were restricted until the sixth postoperative month.

## Result

Table 1 describes the demographic data of the sample, diagnostic specifications, previous surgeries, lesion characteristics, allograft plug size, and follow-up time.

**Table 1:** Demographics of operated knees

Knee	Age at surgery	BMI	Follow-up time (months)	Diagnosis	Previous surgeries	Treated lesion site	Injury severity	Plug size
1	38	25.24	65	OCD/varus	Mosaicplasty and valgus osteotomy	MFC	Grade IV > 4 cm	1 × 25 mm
2	22	24.69	54	OCD	OCD fixation and mosaicplasty	MFC	Grade IV > 4 cm	2 × 22.5 mm
3	21	23.14	49	OCD/varus	Valgus osteotomy	MFC	Grade IV > 4 cm	2 × 22.5 mm
4	22	27.13	44	ON	Valgus osteotomy and partial medial meniscectomy	MFC	Grade IV > 4 cm	2 × 20 mm
5	21	23.14	44	OCD/varus	Valgus osteotomy	MFC	Grade IV > 4 cm	1 × 22.5 mm
6	29	20.81	37	Degenerative injury resulting from PFI	Proximal and distal patellar realignment	Patella	Grade IV 2-4 cm	1 × 20 mm
7	49	22.83	34	Synthetic implant failure	SaluCartilage at trochlea and MFC	MFC and trochlea	Grade IV 2-4 cm	1 × 25 mm / 1 × 22.5 mm
8	29	30.99	10	Chondral lesion from fracture	Tibial bone graft	Lateral plateau	Grade III 2-4 cm	1 × 10 mm in thickness

**Abbreviations:** BMI, body mass index; MFC, Medial femoral condyle; PFI, patellofemoral instability; OCD, osteochondritis dissecans; ON, osteonecrosis.

The age of the patients ranged from 21 to 49 years at the time of surgery. All patients were male. The body mass index (BMI) identified four patients with normal weight, two overweight, and only one with grade-I obesity. The initial diagnoses varied, with four of the eight knees having OCD in their classic location in the MFC. The severity of the treated lesions ranged from grade III to IV according to the ICRS classification, with lesion sizes ranging from 2 to 4 cm in diameter.

Most surgeries occurred in a quaternary hospital of the public healthcare network, followed by a tertiary hospital and a private hospital.

In the follow-up period of up to five years, six of the seven patients who underwent surgery stated that they were satisfied or very satisfied with the procedure. A single patient reported moderate satisfaction with the procedure. He was an older patient with lesions on more than one surgical site (trochlea and MFC).

The functional improvement varied, and out of the seven operated patients, only one presented worsening of the IKDC score during the follow-up period, with a preoperative score of 35.63 and a 1-year postoperative score of 32.1. All remaining subjects presented increased scores, ranging from 32.18 to 64.36 at the final follow-up.

All patients presented quality of life improvement per the KOOS score, ranging from 18.75 to 75 throughout the postoperative follow-up period.

**Fig. 9:**
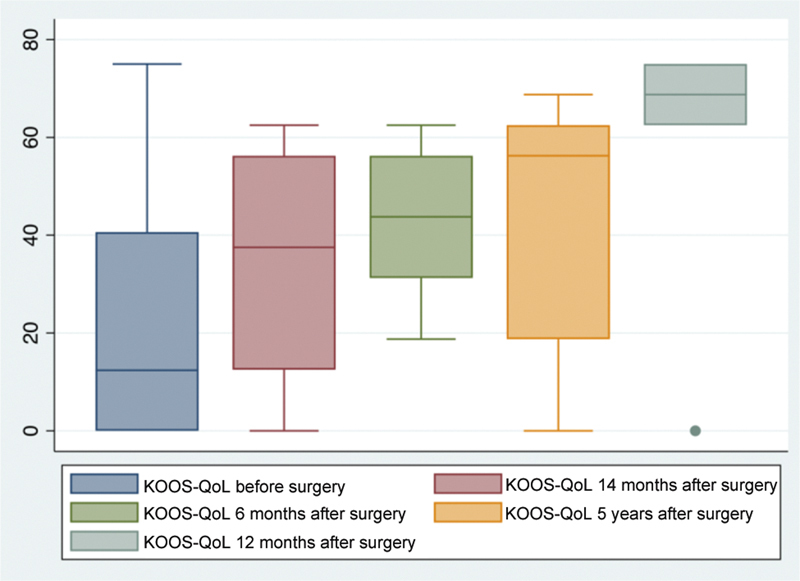
Knee and Osteoarthritis Outcome Score (KOOS), quality-of-life (QoL) item scores preoperatively and 6, 12, 14 months, and 5 years postoperatively.

Figs. 9^10^ , respectively, demonstrate the improvement in the IKDC and KOOS-QoL scores when comparing preoperative values with those at 6, 12, 24 months, and 5 years of postoperative follow-up. The graphs include patients up to the maximum follow-up time corresponding to each period. Table 1 details the follow-up time.

**Fig. 10:**
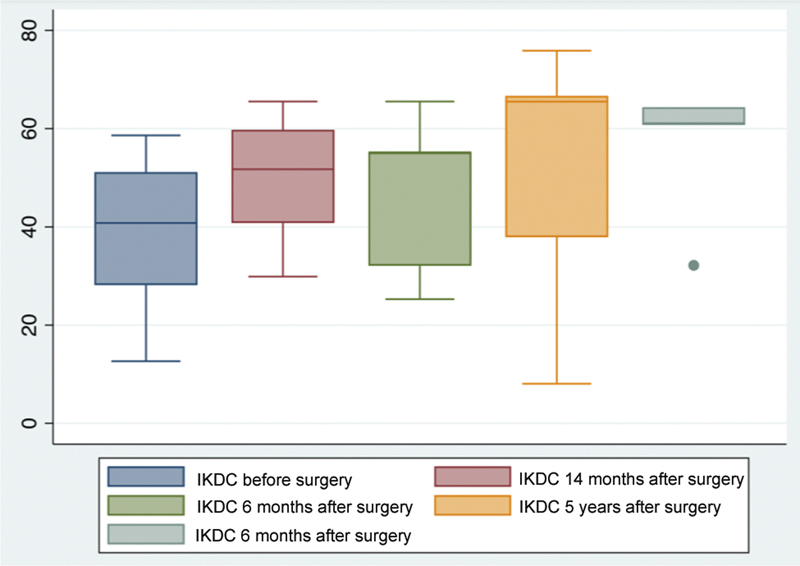
International Knee Documentation Committee (IKDC) scores preoperatively, at 6, 12, 14 months, and 5 years postoperatively.

Table 2 shows a statistically significant improvement in the IKDC score in the follow-up periods of 6 months, 24 months, and 5 years, with values close to 50. The assessment of the KOOS-QL score also showed a statistically significant improvement in 24 months but with lower mean scores.

**Table 2:** Preoperative and postoperative International Knee Documentation Committee (IKDC) and Knee and Osteoarthritis Outcome Score (KOOS) quality of life (QoL) scores with respective p-values.

	6 months	12 months	24 months	5 years
**IKDC**				
Preop	38.93 (±16.04)	41.69 (±12.99)	39.41 (±17.27)	39.54 (±17.28)
Postop	49.99 (±12.27)	47.60 (±14.36)	54.51 (±23.58)	56.54 (±13.72)
* p* -value	0.0497	0.3336	0.0308	0.0073
**KOOS-QoL**				
Preop	22.62 (±27.75)	25.85 (±28.31)	25 (29.09)	28.75 (±32.95)
Postop	34.37 (±24.09)	41.96 (±14.75)	46.42 (±26.23)	56.25 (±31.86)
* p* -value	0.0587	0.0592	0.0413	0.0972

**Abbreviations:** IKDC, International Knee Documentation Committee; KOOS, Knee and Osteoarthritis Outcome Score; Preop, preoperative; postop, postoperative; QoL, quality of life.

There were no failures, need for reintervention, infections, or other complications.

## Discussion

This sample, in a national setting and with the tissue processing available in Brazil, demonstrates that fresh osteochondral homologous transplantation is an adequate method for treating large osteochondral lesions or a salvage method after the failure of a previous procedure. It was a proper method for improving the pain and function of our patients, postponing the future need for total knee arthroplasty in patients who were ineligible for this procedure due to young age.

Fresh homologous osteochondral transplantation is an option deserving of consideration in treating osteochondral lesions. The main indication for this procedure includes symptomatic defects larger than 3 cm in young active patients, as it provides viable hyaline cartilage with metabolically active chondrocytes and subchondral bone with remodeling potential. 11

In addition, it has the advantage of allowing posttransplantation joint load since it provides biologically functional hyaline cartilage surface, making rehabilitation and return to sports easier, 12 improving joint function and symptoms, and delaying arthroplasty. 11

Our results show that transplantation is an alternative treatment, mainly for salvage in patients undergoing failed surgical procedures. An indication for homologous osteochondral transplantation is a revision procedure after failed surgical cartilage restoration. 11

Zouzias and Bugbee 10 report that transplantation warrants consideration as a treatment after other non-surgical treatments have failed. Therefore, its indications include large focal defects, failure of previous cartilage repair, ON, OCD, and posttraumatic osteochondral defects. 11 13 In our study, all cases had undergone previous surgical treatments, and, in half of the subjects, the osteochondral lesions resulted from OCD.

Although our patients had undergone other previous surgical procedures on the knee, we observed a significant posttransplantation improvement in most scores evaluating symptoms, function, and sports activities from IKDC, and improvement in the KOOS-QL score, except for some follow-up times ( Table 2 ).

International Knee Documentation Committee scores improved in six of the seven patients and KOOS-QoL scores improved in all subjects. A single subject reported little satisfaction with the procedure. He was the oldest patient (49 years old) and ineligible for a new approach with arthroplasty. The other patient with no IKDC score improvement at follow-up is the most recently treated subject, with a follow-up time of approximately 10 months. He remains in the rehabilitation phase and had a more complex treatment location due to a fracture of the lateral tibial plateau. 14 We do not consider this case a treatment failure due to the early evolution time for fresh osteochondral transplantation of the lateral plateau.

According to Gracitelli et al., 15 who compared knees undergoing homologous osteochondral transplantation as primary treatment versus treatment after failed subchondral stimulation, prior cartilage repair did not affect the transplant survival and functional outcome.

A systematic review of patients undergoing homologous osteochondral transplantation with a follow-up of at least two years identified a similar clinical improvement in pain, function, and return to sports using the KOOS, Tegner, and Marx scores. 16

This same study reported a high rate of need for surgical reoperation, ranging from 34 to 53% in more than half of the cases due to the presence of loose bodies or for debridement requirements. 16 This contrasts with our study, in which there was no need for injury reoperation or complications. In another study, 13 the reoperation rate was approximately 30% in the first 2 years of follow-up. In our study, there was no need for reoperation in up to 5 and a half years of follow-up.

The changes and improvement in the KOOS-QoL item were not significant, perhaps because of the degenerative and chronic pattern of the patients, who presented a slow return to daily activities.

## Conclusion

The general improvement in symptoms, function, and level of sports activities during follow-up demonstrates that, in knees that have undergone other surgical procedures, fresh homologous osteochondral transplantation leads to good outcomes and improved quality of life.

More studies in our setting are needed with more cases and longer follow-up times to evaluate the potential complications inherent to the procedure, reintervention requirements, and the cost-effectiveness of this technique.

## References

[1] Görtz S Bugbee W D Allografts in articular cartilage repair Instr Course Lect 2007 56 469 480 17472329

[2] Welton K L Logterman S Bartley J H Vidal A F McCarty E C Knee Cartilage Repair and Restoration: Common Problems and Solutions Clin Sports Med 2018 37 02 307 330 29525030 10.1016/j.csm.2017.12.008

[3] Bugbee W D Convery F R Osteochondral allograft transplantation Clin Sports Med 1999 18 01 67 75 10028117 10.1016/s0278-5919(05)70130-7

[4] Gomoll A H Minas T The quality of healing: articular cartilage Wound Repair Regen 2014 22 01 30 38 24813362 10.1111/wrr.12166

[5] Minas T A practical algorithm forcartilage repair Oper Tech Sports Med 2000 8 02 141 143

[6] Langer F Gross A E Immunogenicity of allograft articular cartilage J Bone Joint Surg Am 1974 56 02 297 304 4452688

[7] Czitrom A A Keating S Gross A E The viability of articular cartilage in fresh osteochondral allografts after clinical transplantation J Bone Joint Surg Am 1990 72 04 574 581 2324145

[8] Bugbee W D Pallante-Kichura A L Görtz S Amiel D Sah R Osteochondral allograft transplantation in cartilage repair: Graft storage paradigm, translational models, and clinical applications J Orthop Res 2016 34 01 31 38 26234194 10.1002/jor.22998 PMC4732516

[9] Bugbee W D Fresh osteochondral allografts J Knee Surg 2002 15 03 191 195 12152982

[10] Zouzias I C Bugbee W D Osteochondral Allograft Transplantation in the Knee Sports Med Arthrosc Rev 2016 24 02 79 84 27135291 10.1097/JSA.0000000000000109

[11] Valdivia Zúñiga C A De Cicco F L Osteochondral Allograft. [Updated 2023 Jul 31] Treasure Island (FL) StatPearls Publishing 2023Jan-. Available from:https://www.ncbi.nlm.nih.gov/books/NBK560511/32809346

[12] Patel S Amirhekmat A Le R Williams Iii R J Wang D Osteochondral Allograft Transplantation in Professional Athletes: Rehabilitation and Return to Play Int J Sports Phys Ther 2021 16 03 941 958 34123544 10.26603/001c.22085 PMC8169007

[13] Cavendish P A Everhart J S Peters N J Sommerfeldt M F Flanigan D C Osteochondral Allograft Transplantation for Knee Cartilage and Osteochondral Defects: A Review of Indications, Technique, Rehabilitation, and Outcomes JBJS Rev 2019 7 06 e7 10.2106/JBJS.RVW.18.00123 31220000

[14] Gracitelli G C Tirico L E McCauley J C Pulido P A Bugbee W D Fresh Osteochondral Allograft Transplantation for Fractures of the Knee Cartilage 2017 8 02 155 161 10.1177/1947603516657640 28345414 PMC5358831

[15] Gracitelli G C Meric G Briggs D T Fresh osteochondral allografts in the knee: comparison of primary transplantation versus transplantation after failure of previous subchondral marrow stimulation Am J Sports Med 2015 43 04 885 891 25817190 10.1177/0363546514565770

[16] Crawford Z T Schumaier A P Glogovac G Grawe B M Return to Sport and Sports-Specific Outcomes After Osteochondral Allograft Transplantation in the Knee: A Systematic Review of Studies With at Least 2 Years' Mean Follow-Up Arthroscopy 2019 35 06 1880 1889 31053460 10.1016/j.arthro.2018.11.064

