# Supplementary material for: Assessment of Fresh Homologous Osteochondral Transplantation in Knees as a Salvage Treatment: A Prospective Case Series with a Minimum Follow-up Period of 10 Months
Source: Rev Bras Ortop (Sao Paulo). 2024 Dec 21;59(6):e966–75. doi: 10.1055/s-0044-1792116 (PMC11663053; doi:10.1055/s-0044-1792116)
Supplement: Related texts

# Research article: Avaliação do transplante osteocondral homólogo a fresco no joelho como tratamento de salvação: Série de casos prospectiva com mínimo de dez meses de seguimento

**Keywords:** aloenxertos, cartilagem articular, joelho/cirurgia, osteocondrite, transplante homólogo

## Abstract

**Objetivo** Avaliar o desfecho clínico e a satisfação dos pacientes submetidos ao transplante osteocondral homólogo a fresco no joelho como método de salvação.

**Métodos** Foram analisados no pré-operatório 8 joelhos de 7 pacientes do sexo masculino que foram submetidos ao transplante osteocondral homólogo a fresco por um único cirurgião e acompanhados por um período que variou entre 10 meses e 5 anos e 5 meses. Os desfechos clínicos utilizaram os escores no International Knee Documentation Committee (IKDC) e no item qualidade de vida do Knee and Osteoarthritis Outcome Score (KOOS-QV).

**Resultado** A amostra analisada consistiu em casos complexos, todos com cirurgias prévias nos joelhos operados. A melhora da função variou, sendo que seis dos sete pacientes operados apresentaram melhora clínica estatisticamente significante, de acordo com o escore IKDC, e um único paciente se definiu como moderadamente satisfeito com o procedimento. Todos os pacientes avaliados apresentaram melhora no item qualidade de vida avaliado pelo escore KOOS. Não houve falha, necessidade de reabordagem dos pacientes operados, ou ocorrência de infecção.

**Conclusão** O transplante osteocondral homólogo a fresco é um método de salvação seguro no nosso meio para tratar lesões grandes e com falha de procedimentos prévios. Apesar da pequena amostra desta série de casos, a maioria dos desfechos clínicos foram positivos e sem complicações associadas.

## Introdução

A cartilagem hialina é um tecido avascular, que recebe sua nutrição por meio de difusão do líquido sinovial. 1 Apresenta um potencial de cicatrização limitado, com baixa capacidade regenerativa. 2 Deste modo, lesões de espessura total da cartilagem articular apresentam uma história natural com desfechos ruins, especialmente em indivíduos jovens. 3

Diversos procedimentos reparativos de estímulo da medula óssea já foram utilizados no tratamento de lesões da cartilagem articular, obtendo-se um tecido de fibrocartilagem de qualidade inferior à cartilagem articular 4 com seus resultados sendo ainda mais limitados na presença de lesões grandes (> 3 cm ^2^ ). 5 Em razão disto, o tratamento ideal de uma lesão focal de cartilagem envolve restaurar a estrutura da cartilagem hialina, 2 integrando o tecido à sua periferia e ao osso subcondral 4 gerando a menor morbidade possível e o maior tempo livre de sintomas. 2

É considerado um tecido “imunoprivilegiado,” pois os condrócitos que o compõem se encontram embebidos em matriz acelular, com uma proteção relativa ao sistema imune. 6 7 Somando-se a essas características o fato de ser uma estrutura aneural, torna-se um tecido ideal para o transplante. 1

O transplante osteocondral a fresco é uma técnica que permite tratar lesões extensas de cartilagem e, em um único tempo cirúrgico, restaurar a congruência da superfície articular sem a morbidade do sítio doador. 2 O objetivo deste trabalho é avaliar o desfecho clínico e a satisfação de uma série de casos de pacientes submetidos ao transplante osteocondral homólogo a fresco no joelho como método de salvação com um mínimo de 10 meses de seguimento pós-operatório.

## Materiais e Métodos

Após obter aprovação do Comitê de Ética, identificaram-se sete pacientes, sendo um deles abordado bilateralmente (oito joelhos), que foram submetidos ao transplante osteocondral homólogo a fresco no joelho. Este estudo prospectivo teve um período de seguimento mínimo pós-operatório de 10 meses, e todos os pacientes foram avaliados no pré e pós-operatório por meio dos escores no International Knee Documentation Committee (IKDC) e no item qualidade de vida do Knee and Osteoarthritis Outcome Score (KOOS-QV). Os questionários para avaliação foram aplicados em cada paciente no pré-operatório e, depois, com 6, 12, 24 meses e 5 anos de seguimento pós-operatório.

O pareamento entre o joelho receptor e o doador foi realizado com radiografias nas incidências anteroposterior e perfil, 1 com os tecidos doadores obtidos do banco de tecidos do Instituto Nacional de Traumatologia e Ortopedia (INTO) e do banco de tecidos F. E. Godoy Moreira do Hospital das Clínicas da Faculdade de Medicina da Universidade de São Paulo (HC-FMUSP). Devido à viabilidade celular dos condrócitos no tecido doador, o transplante foi realizado até 4 semanas após a sua captação e preparo, 4 permanecendo refrigerado a uma temperatura de 4° C sem que esse tecido fosse congelado 8 e sem a necessidade de terapia imunossupressora. 9

Foi indicado o transplante osteocondral a fresco para lesões osteocondrais > 2 cm ^2^ de diâmetro, classificadas como grau III ou IV pela International Cartilage Repair Society (ICRS). O diagnóstico das lesões mais comum foi a ostecondrite dissecante (OCD) no côndilo femoral medial (CFM), mas também foram abordados casos de osteonecrose (ON) do CFM, lesão degenerativa na patela por instabilidade femoropatelar (IFP), falha de implante sintético prévio (SaluCartilage - SaluMedica, LLC, Atlanta, GA, EUA) no CFM e tróclea, e lesão condral pós-traumática por fratura do platô tibial lateral.

Todos os pacientes operados nesta série de casos já haviam apresentado falha de tratamentos não-operatório e cirúrgicos prévios no joelho, sendo o transplante osteocondral a fresco proposto como método de salvação para postergar uma eventual necessidade de artroplastia do joelho.

O procedimento foi iniciado após a confirmação da identificação, acondicionamento e qualidade do aloenxerto, sendo este mantido em uma solução salina até seu uso. Quanto à técnica cirúrgica, na maioria dos casos foi realizada artrotomia parapatelar medial. Somente em um caso de transplante do planalto lateral foi feita a via parapatelar lateral estendida. Após exposição articular, realizou-se um desbridamento dos bordos da lesão até que fosse atingido o osso subcondral. O defeito foi mensurado utilizando-se uma ferramenta com moldes cilíndricos (técnica *dowel* ), para determinar o tamanho do aloenxerto a ser captado.

O primeiro caso realizado da série, com um tempo de seguimento de 5 anos e 5 meses está ilustrado a seguir. Trata-se de um paciente do sexo masculino, então com 38 anos, com diagnóstico prévio de OCD que apresentava queixa de dores no joelho esquerdo há 23 anos e já havia sido submetido previamente à mosaicoplastia e meniscectomia parcial, sem melhora significativa.

A Fig. 1 demonstra o exame de ressonância magnética nuclear evidenciando a falha da mosaicoplastia com cistos subcondrais no côndilo femoral medial do joelho esquerdo. As Figs. 2A–D ilustram a radiografia panorâmica antes da osteotomia, com eixo mecânico traçado evidenciando discreto varo, e as radiografias após osteotomia valgizante com cunha de abertura medial e eixo final corrigido.

**Fig. 1:** Sequência de imagens da ressonância nuclear magnética, cortes sagital e coronal, evidenciando falha de integração da mosaicoplastia do côndilo femoral medial e meniscectomia parcial prévia.

**Figs. 2:** Radiografias panorâmicas dos membros inferiores evidenciando eixo mecânico em varo discreto ( **A,B** ). Radiografias anteroposterior e perfil após osteotomia valgizante com cunha de abertura medial e eixo final corrigido ( **C,D** ).

Após realização da artrotomia e identificação da lesão ( Fig. 3A ), foi realizado o desbridamento, regularização e correta medição do defeito ( Figs. 3B,C ), seguido do preparo adequado do aloenxerto com seu plugue osteocondral ( Figs. 3D–F ).

**Figs. 3:** Imagens intraoperatórias mostrando o defeito osteocondral antes do desbridamento ( **A** ), a regularização do defeito com instrumental próprio ( **B** ) e o aspecto após regularização das bordas ( **C** ). Imagem do aloenxerto ( **D** ), retirada do plugue osteocondral com diâmetro apropriado conforme medição prévia ( **E** ) e posterior preparo e lavagem com pulsátil do mesmo a fim de retirar os componentes medulares do osso subcondral ( **F** ).

A localização da retirada do aloenxerto foi estabelecida na tentativa de reproduzir o sítio de lesão dos pacientes, utilizando-se de um fio guia pelo centro do mesmo molde cilíndrico, a fim de centralizar a serra-copo durante a retirada do tecido doador. Mantendo a perpendicularidade à superfície articular, a profundidade do plugue além da cartilagem (no osso subcondral) era de 3 a 4 mm, 1 sendo este mesmo osso subcondral submetido a uma lavagem pulsátil com soro fisiológico para a limpeza de possíveis antígenos provenientes do tecido doador.

A apresentação final após fixação do plugue no intraoperatório está demonstrada nas Figs. 4A,B , seguido pelo exame de ressonância nuclear magnética controle após um ano de seguimento ( Fig. 5 ).

**Figs. 4:** Imagens intraoperatórias antes da fixação ( **A** ) e após fixação do plugue de maneira sob pressão ( **B** ).

**Fig. 5:** Sequência de imagens da ressonância nuclear magnética, cortes sagital e coronal, evidenciando a integração do aloenxerto após 1 ano de pós-operatório. Artefato metálico associado é observado devido matéria de fixação da osteotomia.

Quando a lesão osteocondral apresentava um tamanho maior do que 25 mm de diâmetro, optava-se pela retirada de mais de um plugue do aloenxerto, com subsequente sobreposição de suas áreas (“Técnica boneco de neve” / “ *snowman technique* ”). 1 A fixação do tecido doador foi feita sob pressão ( *press-fit* ) e, quando se apresentava instável, optou-se por adicionar um parafuso de Hebert no centro de cada plugue “instável” para agregar maior estabilidade.

No segundo caso demonstrado a seguir, foi necessária a realização desta maneira, com a utilização de dois plugues no joelho esquerdo devido à extensão da lesão. Trata-se de paciente do sexo masculino, então com 21 anos, com OCD bilateral e fragmento osteocondral instável e solto na articulação ( Fig. 6 ). Apresentava geno varo bilateral, sendo submetido à osteotomia valgizante em dois tempos.

**Fig. 6:** Sequência de imagens da ressonância nuclear magnética, cortes coronais, evidenciando a lesão no côndilo femoral medial do joelho direito e joelho esquerdo, respectivamente, por osteocondrite dissecante.

As imagens intraoperatórias do transplante osteocondral estão ilustradas na Fig. 7 . O tecido osteocondral a fresco doador e seu preparo e fixação no joelho esquerdo com dois plugues, sendo um fixado sob pressão e o segundo com parafuso sem cabeça metálico estão demonstrados ( Figs. 7A–D ), bem como o preparo cirúrgico no joelho direito, realizado 5 meses após a cirurgia no joelho esquerdo ( Figs. 7E,F ). A imagem radiográfica pós-operatória ( Fig. 8A ) e ressonância com 10 meses pós cirurgia ( Fig. 8B ) demonstram artefatos metálicos, porém boa integração do aloenxerto e superfície condral uniforme foi observada.

**Figs. 7:** Tecido osteocondral a fresco do côndilo femoral medial ( **A** ). Foto intraoperatória do joelho esquerdo durante execução do transplante a fresco com 2 plugs osteocondrais de 22,5 mm cada ( **B,C** ) e fixação do plug inferior com parafuso sem cabeça e o plug superior fixado com a técnica sob pressão ( **D** ). Foto intraoperatória do joelho direito durante execução do transplante a fresco do côndilo femoral medial com 1 plug osteocondral de 22,5 mm fixado de maneira sob pressão ( **E–F** ).

**Figs. 8:** Radiografia pós-operatória após transplante osteocondral bilateral com osteotomias consolidadas (operados com diferença de 5 meses) ( **A** ). Imagens de ressonância nuclear magnética pós-operatória do joelho direito ponderada em T1 com artefatos metálicos após 10 meses da cirurgia. As imagens demonstram boa integração e superfície condral adequada ( **B** ).

No pós-operatório, seguiu-se protocolo de reabilitação para proteção do enxerto enquanto o mesmo incorporava-se à área receptora. 10 Os pacientes foram mantidos durante 6 semanas sem aplicação de carga no membro operado, com o uso de imobilizador longo articulado de joelho em extensão até controle da ativação de quadríceps. Impôs-se uma restrição de arco de movimento até 90° nas primeiras 4 semanas, sendo estimulada isometria de quadríceps desde o pós-operatório imediato e exercícios de cadeia cinética fechada após a 6ª semana pós-operatória. Mantiveram-se restrições quanto ao retorno a atividades esportivas até o 6° mês pós-operatório.

## Resultado

Os dados demográficos da amostra analisada, especificações diagnósticas, cirurgias prévias, características das lesões, tamanho dos plugues de aloenxerto e tempo de seguimento são descritos na Tabela 1 .

**Tabela 1:** Perfil demográfico dos joelhos operados

Joelho	Idade na cirurgia	IMC	Tempo de seguimento em meses	Diagnóstico	Cirurgia prévia	Localização das lesões tratadas	Grau da lesão	Tamanho dos plugs
1	38	25,24	65	OCD/VARO	Mosaicoplastia e osteotomia valgizante	CFM	Grau IV > 4 cm	1 × 25 mm
2	22	24,69	54	OCD	Fixação OCD e Mosaicoplastia	CFM	Grau IV > 4 cm	2 × 22,5 mm
3	21	23,14	49	OCD/VARO	Osteotomia Valgizante	CFM	Grau IV > 4 cm	2 × 22,5 mm
4	22	27,13	44	ONC	Osteotomia Valgizante e meniscectomia parcial medial	CFM	Grau IV > 4 cm	2 × 20 mm
5	21	23,14	44	OCD/VARO	Osteotomia Valgizante	CFM	Grau IV > 4 cm	1 × 22,5 mm
6	29	20,81	37	Lesão degenerativa por IFP	Realinhamento patelar proximal e distal	Patela	Grau IV 2-4 cm	1 × 20 mm
7	49	22,83	34	Falha de implante sintético	Salucartilagem na tróclea e CFM	CFM e tróclea	Grau IV 2-4 cm	1 × 25 mm / 1 × 22,5 mm
8	29	30,99	10	Lesão condral por fratura	Enxerto ósseo na tíbia	Platô lateral	Grau III 2-4 cm	1 × 10 mm espessura

**Abreviaturas:** CFM, côndilo femoral medial; IMC, índice de massa corporal; IFP, instabilidade femoropatelar; OCD, osteocondrite dissecante; ONC, osteonecrose.

A idade dos pacientes, em sua totalidade do sexo masculino, variou entre 21 e 49 anos no momento da cirurgia. O índice de massa corpórea (IMC) apresentado identificou quatro pacientes com peso normal, dois com sobrepeso e apenas um com obesidade grau I. Os diagnósticos iniciais variaram, sendo que, quatro dos oito joelhos avaliados, apresentavam como diagnóstico base a OCD, com sua localização clássica no CFM. O grau das lesões tratadas variou de III a IV pela classificação ICRS, com o tamanho das lesões variando de 2 a 4 cm de diâmetro.

As cirurgias foram, em sua maioria, realizadas em hospital quaternário da rede pública de saúde, um hospital terciário e um hospital privado.

No seguimento de até cinco anos, seis dos sete pacientes operados declararam-se satisfeitos ou muito satisfeitos com a cirurgia realizada, com apenas um paciente pouco satisfeito com o procedimento ao qual foi submetido, tratando-se de paciente com idade mais avançada e lesões tratadas em mais de um sítio cirúrgico (tróclea e CFM).

A melhora da função variou, sendo que dos 7 pacientes operados, apenas 1 apresentou, no seguimento, piora do escore IKDC, com escore pré-operatório de 35,63 e pós-operatório de um ano de 32,1. Todos os outros apresentaram aumento do valor do escore, com variação de valores entre 32,18 e 64,36 no seguimento final.

Todos os pacientes analisados apresentaram melhora do escore KOOS-QV, com uma variação entre 18,75 e 75 ao longo do seguimento pós-operatório.

As Figs. 9 10 demonstram, respectivamente, a melhora dos escores IKDC e KOOS-QV quando comparados os valores pré-operatórios com aqueles aos 6, 12, 24 meses e 5 anos de seguimento pós-operatório. Os gráficos incluem os pacientes até o tempo de seguimento máximo correspondente de cada período. O tempo de seguimento é detalhado na Tabela 1 .

**Fig. 9:** Análise dos scores no item de qualidade de vida do Knee and Osteoarthritis Outcome Score (KOOS-QV) no pré-operatório, com 6, 12 ,14 meses e 5 anos de pós-operatório.

**Fig. 10:** Análise dos escores no International Knee Documentation Committee (IKDC) no pré-operatório, com 6, 12 ,14 meses e 5 anos de pós-operatório.

Conforme demonstrado na Tabela 2 , os valores do escore IKDC tiveram uma melhora estatisticamente significante nos períodos de seguimento de 6 meses, 24 meses e 5 anos, mantendo-se com valores próximos a 50. Já a avaliação do escore KOOS-QV também apresentou melhora estatisticamente significativa no período de 24 meses, porém com valores menores nos escores médios.

**Tabela 2:** Escores pré e pós-operatórios do IKDC e KOOS qualidade de vida (QV) com valor de p respectivo

	6 meses	12 meses	24 meses	5 anos
**IKDC**				
Pré-op.	38,93 (±16,04)	41,69 (±12,99)	39,41 (±17,27)	39,54 (±17,28)
Pós-op.	49,99 (±12,27)	47,60 (±14,36)	54,51 (±23,58)	56,54 (±13,72)
Valor de *p*	0,0497	0,3336	0,0308	0,0073
**KOOS-QV**				
Pré-op.	22,62 (±27,75)	25,85 (±28,31)	25 (29,09)	28,75 (±32,95)
Pós-op.	34,37 (±24,09)	41,96 (±14,75)	46,42 (±26,23)	56,25 (±31,86)
Valor de *p*	0,0587	0,0592	0,0413	0,0972

**Abreviaturas:** IKDC, International Knee Documentation Committee; KOOS, Knee and Osteoarthritis Outcome Score; Preop, pré-op., pré-operatório; pós-op., pós-operatório; QV, qualidade de vida.

Não houve falha ou necessidade de reabordagem dos pacientes operados, bem como relato de infecção ou outras complicações.

## Discussão

Esta amostra em ambiente nacional e com os processamentos de tecido disponíveis no Brasil demonstra o transplante homólogo osteocondral a fresco como método adequado para tratamento de lesões osteocondrais grandes ou como método de salvação após falha de procedimento prévio assim como para a melhora da dor e função dos pacientes nesta amostra seralém de capaz de postergar a necessidade futura de artroplastia total do joelho em pacientes inelegíveis para artroplastia devido à pouca idade.

O transplante osteocondral homólogo a fresco é uma opção que deve ser considerada no tratamento das lesões osteocondrais. É indicado principalmente para defeitos sintomáticos maiores que 3 cm em pacientes jovens ativos pois fornecem cartilagem hialina viável com condrócitos metabolicamente ativos e osso subcondral com potencial de remodelação. 11

Além disso, apresenta a vantagem de permitir carga na articulação após o transplante por ser capaz de promover uma superfície de cartilagem hialina biologicamente funcional, tornando a reabilitação e o retorno aos esportes mais fácil 12 e melhorando, portanto, a função articular e os sintomas e retardando a artroplastia. 11

Com os resultados apresentados, verificamos que o transplante é uma alternativa a ser usada principalmente em casos de salvação, em que o paciente já foi submetido a outros procedimentos cirúrgicos e houve falha. Uma das indicações de transplante osteocondral homólogo é seu uso para revisão de procedimentos nos cases em que outras técnicas cirúrgicas de restauração da cartilagem não funcionaram. 11

De acordo com Zouzias e Bugbee, 10 o transplante somente deve ser considerado como tratamento após falha de outros tratamentos não cirúrgicos. É indicado, portanto, em grandes defeitos focais, falha de reparo prévio da cartilagem, casos de osteonecrose, osteocondrite dissecante e/ou defeitos osteocondrais pós-traumáticos. 11 13 No presente estudo, todos os casos analisados já haviam sido submetidos a tratamentos cirúrgicos anteriores e, na metade dos casos, as lesões osteocondrais foram causadas por OCD.

Embora na série de casos analisada os pacientes já tivessem sido submetidos a outros procedimentos cirúrgicos prévios no joelho, foi possível identificar que, após a realização do transplante, houve uma melhora significativa da maioria dos escores que avaliam sintomas, função e atividades desportivas através do IKDC, bem como melhora no escore KOOS-QV, exceto para alguns tempos de seguimento ( Tabela 2 ).

A avaliação dos escores IKDC demonstrou que houve melhora em seis dos sete pacientes analisados e em todos os pacientes operados pelo escore KOOS-QV. A declaração de pouco satisfeito com o procedimento foi feita por apenas um paciente, tratando-se do paciente com idade mais avançada (49 anos) e inelegível para uma nova abordagem com artroplastia. O outro paciente que não apresentou melhora no score IKDC ao longo do seguimento corresponde ao paciente mais recentemente abordado, com tempo de seguimento aproximado de dez meses, ainda em fase de reabilitação e numa localização mais complexa de tratamento devido a uma fratura do planalto tibial lateral. 14 Neste caso não consideramos falha de tratamento devido ao tempo de evolução ainda precoce para o transplante osteocondral a fresco do planalto lateral.

De acordo com Gracitelli et al., 15 que comparou joelhos submetidos ao transplante osteocondral homólogo como tratamento primário versus tratamento após falha em joelhos já submetidos ao estímulo subcondral, o reparo prévio da cartilagem não afetou a sobrevida e o resultado funcional do transplante.

Essa mesma melhora foi identificada numa revisão sistemática com pacientes submetidos ao transplante osteocondral homólogo com seguimento de, no mínimo, dois anos, na qual identificou-se melhora clínica da dor, função e retorno ao esporte avaliados pelos escores KOOS, Tegner e Marx. 16

Nesse mesmo estudo, a taxa de necessidade de reabordagem cirúrgica foi alta, variando entre 34 e 53% em mais da metade dos casos, por presença de corpos livres ou necessidade de desbridamento, 16 ao contrário do que mostrou nosso estudo, no qual não houve necessidade de reabordagem das lesões ou complicações. Em outro estudo, 13 a taxa de reoperação foi de aproximadamente 30% nos primeiros 2 anos de seguimento. Em nosso estudo, em até 5 anos e meio de seguimento, não houve necessidade de nova reabordagem.

As alterações e melhora no item KOOS-QV não se mostraram significativas, talvez pelo padrão degenerativo e crônico dos pacientes que apresentaram perfil lento de retorno às atividades diárias.

## Conclusão

A melhora geral nos sintomas, função, e nível das atividades desportivas ao longo do seguimento, demonstram que, no joelho já submetido a outros procedimentos cirúrgicos, o transplante osteocondral homólogo a fresco leva a bons resultados e melhora da qualidade de vida.

Mais estudos em nosso meio são necessários com maior número de casos e tempo de seguimento, a fim de avaliarmos as possíveis complicações inerentes ao procedimento e a necessidade de possível reabordagem, bem como custo-efetividade do procedimento analisado.
